# Improved electrical transport properties of an n-ZnO nanowire/p-diamond heterojunction

**DOI:** 10.1039/c8ra03546f

**Published:** 2018-08-13

**Authors:** Dandan Sang, Qingru Wang, Qinglin Wang, Dong Zhang, Haiquan Hu, Wenjun Wang, Bingyuan Zhang, Quli Fan, Hongdong Li

**Affiliations:** School of Physics Science and Information Technology, Shandong Key Laboratory of Optical Communication Science and Technology, Liaocheng University Liaocheng 252059 China wangqinglin@lcu.edu.cn phywwang@163.com; State Key Laboratory of Superhard Materials, Jilin University Changchun 130012 China; Key Laboratory for Organic Electronics & Information Displays, Institute of Advanced Materials, Nanjing University of Posts & Telecommunications Nanjing 210046 China

## Abstract

A heterojunction of n-ZnO nanowire (NW)/p-B-doped diamond (BDD) was fabricated. The rectifying behavior was observed with the turn on voltage of a low value (0.8 V). The forward current at 5 V is 12 times higher than that of a larger diameter n-ZnO nanorod (NR)/p-BDD heterojunction. The electrical transport behaviors for the comparison of n-ZnO NWs/p-BDD and n-ZnO NRs/p-BDD heterojunctions are investigated over various bias voltages. The carrier injection process mechanism for ZnO NWs/BDD is analyzed on the basis of the proposed equilibrium energy band diagrams. The ZnO NWs/BDD heterojunction displays improved *I*–*V* characteristics and relatively high performance for the electrical transport properties.

## Abstract 2

A heterojunction of n-ZnO nanowire/p-diamond was fabricated and exhibits improved electrical transport properties, which offer a promising design for developing optoelectronic nanodevices working at the nano-scale and with severe environments.

## Graphical abstract

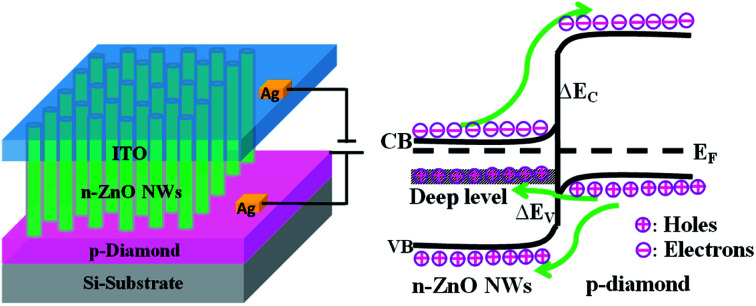

## 1. Introduction

Zinc oxide is a suitable semiconductor because of its promising application in photodetectors,^1–3^ light-emitting diodes (LED),^4^ and solar cells^5^ on account of its wide bandgap (3.37 eV) and high electron mobility at room temperature.^6^ Multiple patterns of ZnO nanostructures, for example (*e.g.*) nanowires,^7^ nanorods,^8^ nanobelt,^9^ nanosheets^10^ have been extensively researched because of their excellent photoelectric properties compared to the bulk. Among the nanostructures above, ZnO nanowires (NWs) are more attractive due to their better carrier confinement and high density surface trap states,^11^ which can enhance the electrical properties and ultraviolet (UV) photodiodes performance of the devices.^12,13^ However, in virtue of the deep acceptor impurity states, low solubility dopants and the self-compensation effect, the intrinsically n-type semiconductor ZnO is difficult to manufacture with a p–n homo-junction architecture for many electronic and optical devices.^14^ Thus far, additional p-type substrate materials, for instance, Si,^15^ Cu_2_O,^16^ GaN,^17^ graphene,^18^ NiO,^7^ B-doped diamond film (BDD)^19–22^ and organic materials,^23^ have been exploited as the candidate to replace p-type ZnO semiconductors for the fabrication of pn heterojunctions. Among them, the B-doped diamond film (bandgap 5.47 eV) is appropriate as a high-temperature p-type conductive material. Up to the present, the electrical transport behavior at higher temperature,^21^ the ultraviolet photoelectrical properties^19,24^ and the photocatalytic activities^25,26^ of n-ZnO NRs/p-diamond heterojunctions have been investigated, however, the n-ZnO NRs/p-BDD heterojunctions have low forward current with 20–80 μA at 5 V.^20,25,27^

In this work, we fabricated n-ZnO NWs/p-BDD heterojunction and obtained better *I*–*V* performance with forward current of 600 μA. We have made a detail analysis of the current–voltage (*I*–*V*) performance of 1D n-ZnO NWs/p-diamond heterojunction. The heterojunction parameters (the turn on voltage, the ideality factor and reverse leakage current *etc.*) and the electrical transport properties have been explored and discussed thoroughly for 1D n-ZnO NWs/p-diamond. The electrical properties of the 1D n-ZnO NWs/p-diamond heterojunction are significantly enhanced compared to the fabricated n-ZnO nanorods (NRs)/p-diamond heterojunction.

## 2. Experimental

Prior to the BDD growth, the hydrogen termination (111) oriented single crystal silicon wafer (1 cm × 1 cm) was immersed into the ethanol and acetone for 5 minutes ultrasonic cleaning, respectively. Then the p-type BDD film with a thickness of ∼4 μm was synthesized on the silicon wafers by chemical vapor deposition (CVD) method. The as grown ZnO NWs and NRs on BDD film was performed by hydrothermal approach. Prior to the hydrothermal procedure, ZnO seed layer with a thickness of ∼30 nm was grown by magnetron sputtering. 15 mL zinc acetate dehydrate (Zn(CH_3_COOH)_2_·2H_2_O) and 15 mL hexamethylenetetramine (HMT·C_6_H_12_N_4_) aqueous solution with 0.05 M for NWs and 0.1 M for NRs concentration were compound together, then maintain 10 min under slight magnetic mixing. The particular of the BDD, ZnO NWs and NRs deposited parameters can be discovered in our former work.^18,22^

The morphologies and structures aspects of the as-fabricated ZnO and diamond were detected by scanning electron microscope (JEOL JXA-8200), the transmission electron microscopy (TEM) images were taken using a JEM-2100 transmission electron microscope with an operating voltage of 200 kV. Raman microscopy (514.5 nm line of an Ar^+^ ion laser), and X-ray diffraction (Rigaku D/MAX-RA with Cu K_α_ radiation of *l* = 1.54056 Å). The absorption spectra were analyzed by UV-Vis absorption spectroscopy (UV-3150 spectrophotometer). The *I*–*V* curves of the heterojunctions were tested by a Keithley 2400 source.

## 3. Results and discussions

**Fig. 1:**
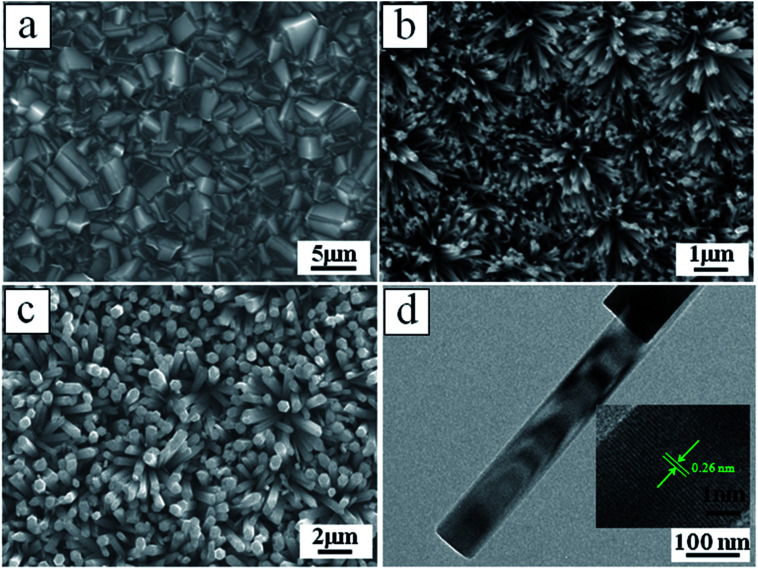
(a) SEM image with plane vertical view of BDD substrate, (b) SEM image of ZnO NWs deposited on the BDD substrates, (c) SEM image of ZnO NRs deposited on the BDD substrates, (d) TEM image of ZnO NWs, inset shows a typical HRTEM image of ZnO NWs.

**Fig. 2:**
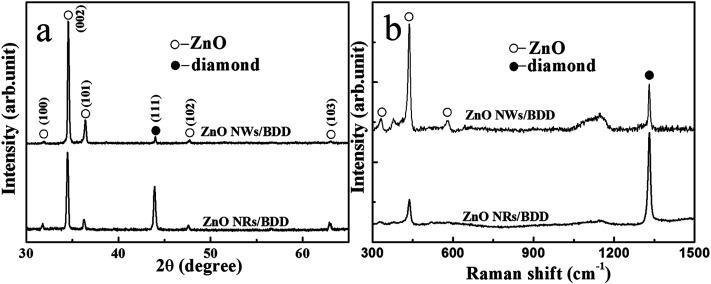
(a) XRD and (b) Raman pattern of ZnO NWs and ZnO NRs on BDD substrates, respectively.

SEM image displays the CVD BDD film with uniform grains by average diameter of ∼2 μm (Fig. 1(a)). The typical vertical-view SEM image of ZnO NWs and ZnO NRs are presented in Fig. 1(b) and (c), respectively. It is found that the ZnO NWs and NRs are generally grown vertically-aligned on the diamond grain planes. The average diameters and lengths of ZnO NWs (NRs) are 80 nm (600 nm) and 500 nm (2 μm), respectively. It is obvious that the diameter of the ZnO NWs is thinner than the ZnO NRs structure. Observed from the TEM (Fig. 1(d)), the ZnO NWs have a large aspect ratio with a smooth surface. Inset shows a typical HRTEM image of ZnO NWs which reveals that the growth of ZnO NWs are along the [0001] direction and the d-spacing was obtained to be 2.60 Å corresponding to the (100) planes of hexagonal ZnO. The ZnO NWs and NRs crystalline structures deposited on BDD substrates were investigated by XRD (Fig. 2(a)) and Raman microscopy (Fig. 2(b)) measurements. As shown of XRD pattern in Fig. 2(a), in addition to the diamond (111) facet diffractive peaks presented at 43.9°, the other main peaks are indexed to the (100), (002), (101), (102), and (103) diffractions peaks from ZnO hexagonal structure. In the Raman pattern (Fig. 2(b)), the 1332 cm^−1^ peak originates from the diamond intrinsic zone-centre phonon band,^28^ and the 332 cm^−1^, 437 cm^−1^ and 582 cm^−1^ peaks are indexed for the E_2H_–E_1H_, E_1H_ and A_1_(LO) mode of ZnO, respectively.^29^ The XRD and Raman spectrum indicate the fabricated ZnO NWs and NRs deposited on the BDD substrate are with single crystal and mostly along the [0001] orientation.

**Fig. 3:**
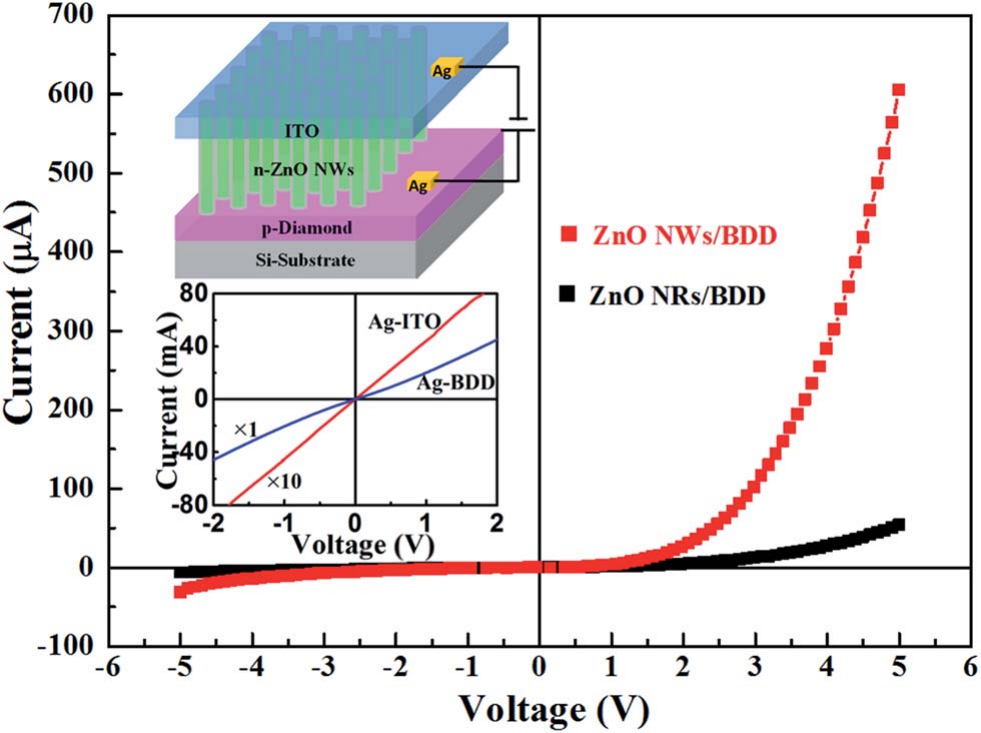
The *I*–*V* plots of the n-ZnO NWs/p-BDD and n-ZnO NRs/p-BDD heterojunction, respectively. The top inset is the schematic of n-ZnO NWs/p-BDD heterojunction device; the bottom inset is linear relation ohmic contacts measurements for Ag/BDD and Ag/ZnO.

The schematic of n-ZnO NWs/p-BDD heterojunction devices are depicted in top inset of Fig. 3. Two silver (Ag) wires are connected on BDD wafer as the positive electrodes. The n-type ZnO pressed with ITO is negative electrodes. The *I*–*V* performances of Ag/BDD and Ag/ITO both exhibits linear relation (bottom inset of Fig. 3) indicating the ohmic contact. In addition, ZnO-ITO is of ohmic contact attributed to the nearly similar work function of the two structure.^30^ Tested by Hall-effect instrument, the carrier density, the resistivity, and the mobility of the BDD is 1.5 × 10^18^ cm^−3^, 0.11 Ω cm, and 39.4 cm^2^ V^−1^ s^−1^, respectively.

The *I*–*V* plots of the n-ZnO NWs/p-BDD and n-ZnO NRs/p-BDD heterojunction were shown Fig. 3. It displays well rectification properties with a rectify ratio of 19.3 and 7.8 at 5 V for the two heterojunction, respectively. The forward turn on voltage is 1.7 V for n-ZnO NRs/p-BDD heterojunctions, and decreased to 0.8 V for ZnO NWs/BDD. The turn on voltage fall to a lower value for ZnO NWs/BDD confirming a good quality interface properties. The reason for this is mainly owing to the generation of more free charge electrons in ZnO NWs to the heterojunction compared to ZnO NRs.^31^ Moreover, the turn-on voltage for the n-ZnO NWs/p-BDD device in this work is 10 times lower than the reported n-ZnO NWs/p-NiO heterojunction^32^ and 6 times lower than the n-ZnO NWs/p-Zn_2_GeO_4_ heterojunction.^33^ Importantly, compared to the fabricated n-ZnO NRs/p-BDD heterojunction, the larger rectification ratio and the lower turn on voltage of n-ZnO NWs/p-BDD suggest that the latter has relatively high performance.

The reverse leakage current is 30.47 μA and 6.45 μA at reverse bias of −5 V for ZnO NWs/BDD and ZnO NRs/BDD, respectively. The larger leakage current for ZnO NWs/BDD was mostly attributed to tunneling effect induced by the more trap centers and higher defect concentration from the interface compared to ZnO NRs/BDD heterojunction.^31^ It is noted that the forward current of ZnO NWs/BDD heterojunction is significantly increased. At the forward voltage of +5 V, the current is 607.2 μA, which is more than 12 times higher than the ZnO NRs/BDD heterojunction (52.7 μA). In virtue of the existence of more surface trap states of deep level in the 1D ZnO NWs,^11^ the ZnO NWs/BDD heterojunction with larger forward current thus exhibits improved *I*–*V* characteristics and better electrical transport properties compared to ZnO NRs/BDD heterojunction.

**Fig. 4:**
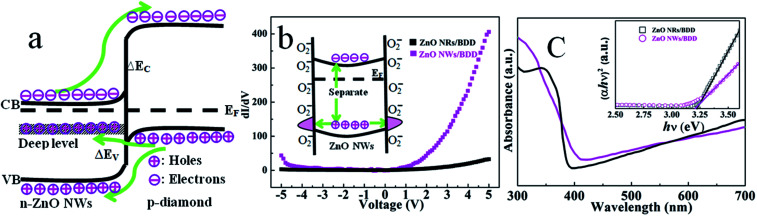
(a) The energy band structure of the ZnO NWs/BDD heterojunction. (b) The plots of d*I*/d*V* with the voltage of the ZnO NWs/BDD and ZnO NRs/BDD devices, respectively. (c) UV-Vis absorption spectrum of the ZnO NWs and ZnO NRs grown on BDD.

To deep-understand the mechanism of reduction of the turn on voltage and enlargement of the forward current for-ZnO NWs/BDD heterojunction, we propose a schematic energy band diagram at thermal equilibrium (Fig. 4(a)). As the electron barrier Δ*E*_c_ is two times larger than the hole barrier Δ*E*_v_, the injection current is mainly caused by the injected holes from the valence band.^20,21^ Compared to ZnO NRs, the ZnO NWs presented more defect-level including deep levels, which are generally originate from the surface states oxygen vacancies and zinc interstitials.^34^ When the forward current is increased, the ZnO NWs deep levels band and bandgaps move up, the more injected holes from diamond valence band can tunnel to the deep levels of the ZnO NWs, forming a larger tunneling current. However, there exists less tunneling effect for ZnO NRs/BDD heterojunction. In addition, the as grown ZnO NWs possess more surface defects compared to ZnO NRs (inset in Fig. 4(b)). The higher density of oxygen from ambient air related surface trap states will adhere to the surface of ZnO NWs and act as trap centers. Then ZnO NWs can make the more negatively O_2_ charged, and produce a built-in barrier on the surface ZnO NWs. After the forward voltage is applied, thermal emission electron hole pairs are produced. The absorbed oxygen trap centers will capture more holes, leading to the separation and diffusion of the electron–hole pairs. The built-in barrier would prevent the electron hole pair's recombination and prolong the life of thermal emission carriers, resulting in much more free electrons injected from ZnO NWs conduction band to the diamond conduction band. Moreover, UV-Vis absorption spectra (Fig. 4(c)) were examined to further analyze the band gaps of the ZnO NWs and ZnO NRs. The band gap for ZnO NWs and NRs can be determined by the following formula,^35^*αhν* = *D*(*hν* − *E*_g_)where *α* is the absorption coefficient, *hν* is the photon energy, *D* is a constant, and *E*_g_ is the optical band gap. The variations of (*αhν*)^2^*versus* the photon energy *hν* in the fundamental absorption region are plotted in the inset of Fig. 4(c). The *E*_g_ values are estimated to be 3.20 eV and 3.23 eV for ZnO NWs and NRs, respectively, by extrapolating the linear portion to the photon energy axis. The lower value of *E*_g_ for ZnO NWs leads to lower hole barrier Δ*E*_v_, and more holes were injected from diamond valence band to ZnO valence band. As a result, the forward current is larger in forward bias scale and the turn on voltage is lowered for ZnO NWs/BDD heterojunction.

Differential conductivity (d*I*/d*V*) is the derivative of the *I*–*V* plots to obtain conduction properties in the heterojunction for it is directly proportional to the states density. Fig. 4(b) shows the variation plots in d*I*/d*V* with the applied bias voltage for both of ZnO NWs/BDD and ZnO NRs/BDD heterojunctions. For the ZnO NRs/BDD heterojunction, the d*I*/d*V* plots are almost constant as a result of the less charge carrier concentrations. However, for ZnO NWs/BDD heterojunction the d*I*/d*V* plots increases with the applied bias voltage, indicating an increase free charge carriers. Therefore, more oxygen trap centers in ZnO NWs/BDD heterojunction capture more holes, leading to an increase of free carriers which make an important contribution to the higher injected current.

**Fig. 5:**
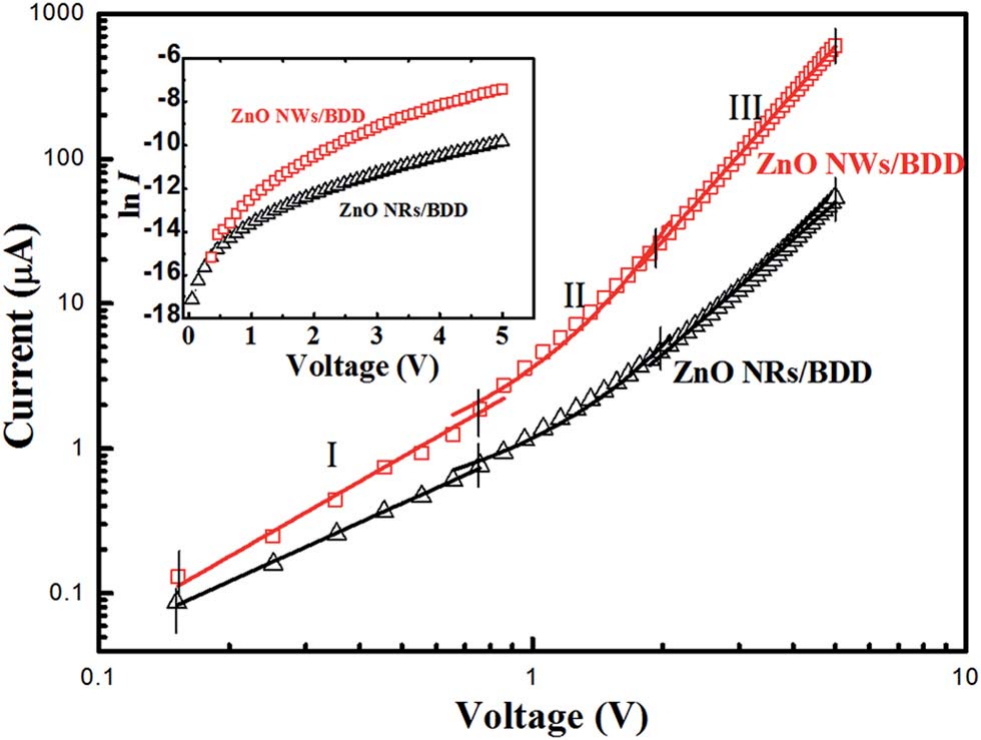
log *I*–log *V* scale plots of the ZnO NWs/BDD and ZnO NRs/BDD heterojunctions, respectively (inset: the curve of the ln *I*–*V*).

Semilog plots of *I*–*V* characteristics are in the inset of Fig. 5 for ZnO NWs/BDD and ZnO NRs/BDD heterojunctions. According to the thermionic emission model equation,
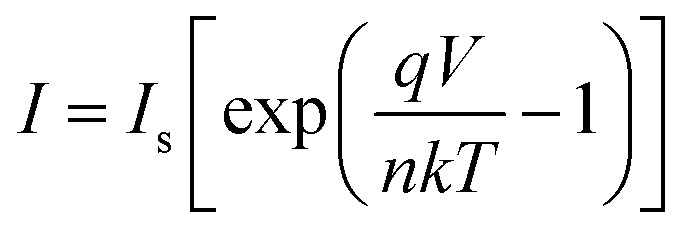
where the pre-exponential factor *I*_s_ is the reverse saturation current. The ideality factor *n* for the ZnO NWs/BDD and ZnO NRs/BDD heterojunctions is estimated to be about 6.2 and 8.6, respectively. The *n* value larger than 2 indicates deviating from ideal pn heterojunction.^36^ Although *n* values are higher than 2, it is much better than the reported ZnO NRs/Si (12.8)^31^ and ZnO NWs/Zn_2_GeO_4_ heterojunctions.^33^ This can be result from the existence of surface states and tunneling induced by deep level between ZnO nanostructures and diamond substrate. The *n* value for ZnO NWs/BDD heterojunction is smaller than the ZnO NRs/BDD heterojunction which can be explained as follows: compared to ZnO NRs/BDD heterojunction, more carriers were existed in ZnO NWs/BDD heterojunction, and then the tunneling effect and the carrier generation-recombination procedure were emerging in the depletion region of the ZnO NWs/BDD heterojunction.

The log *I*–log *V* scale plots of the ZnO NWs/BDD and ZnO NRs/BDD heterojunctions are shown in Fig. 5. Both the two plots were separated into three different regions. At low voltage range (depicted region I, *V* < 0.75 V), the current transmission (*I*–*V*) follows a linear relation of ohmic law. For the moderately voltage of region II (0.75 V < *V* < 2 V), the forward current is following exponential regular increase and obeys a *I*–exp(*αV*) relationship, that is generally obtained from wide bandgap semiconductor heterojunction device results from the recombination tunneling principle.^37^ By fitting the curve of region II, the injection efficiency constant *α* is calculated to be 2.2 V^−1^ and 1.5 V^−1^ for the ZnO NWs/BDD and ZnO NRs/BDD heterojunction, respectively. Both values are close to 1.5 V^−1^, which indicates the fabricated ZnO NWs/BDD and ZnO NRs/BDD heterojunctions are ideal vacuum diodes.^38^ Moreover, a larger *α* indicates a higher thermal excited carrier injection.^31^ In this region, compared to ZnO NRs/BDD, the enlargement of *α* for ZnO NWs/BDD heterojunction can origin from the much more thermally emission carriers (electrons) induced by the more surface states, which fill the unoccupied traps.

When the forward bias voltage is higher than 2 V for region III (2 V < *V* < 5 V), the current transport properties according with the law of *I*–*V*^3.4^ and *I*–*V*^2.6^ for the ZnO NWs/BDD and ZnO NRs/BDD heterojunctions, respectively. The exponents value are higher than 2 normally ascribed to the space-charge-limited current (SCLC) transmission model.^39,40^ The high density trap states from the oxygen surface defect in the ZnO NWs/BDD heterojunction would capture thermal emission carriers (holes), leading to the jump of electrons into the diamond conduction band more easily. Therefore, in region III, the injected current obeys the trap-free SCLC mechanism with larger exponential than ZnO NRs/BDD heterojunction.

## 4. Conclusions

In summary, a heterojunction of n-ZnO NWs/p-BDD diode device have been fabricated. The larger tunneling current from diamond valence band to the deep levels of the ZnO NWs and more oxygen trap centers in ZnO NWs lowered the turn on voltage of the n-ZnO NWs/p-BDD heterojunction and promote the rectification ratio, whereas the forward current at 5 V is 12 times higher. It is proposed that the ZnO NWs/BDD heterojunction exhibits overall improved *I*–*V* characteristics and electrical transport properties with relatively high performance. The fabricated ZnO NWs/BDD heterojunction offers a promising design with a high performance for the developing of optoelectronic nanodevice, especially working at nano-scale and severe environments.

## Conflicts of interest

There are no conflicts to declare.

## Supplementary Material
